# Deferral of non-emergency cardiac interventions is associated with increased emergency hospitalizations up to 24 months post-procedure

**DOI:** 10.1007/s00392-024-02380-y

**Published:** 2024-03-06

**Authors:** Stefanie Andreß, Dominik Felbel, Dominik Buckert, Wolfgang Rottbauer, Armin Imhof, Tilman Stephan

**Affiliations:** Department of Cardiology, Angiology, Pneumology, and Intensive Care Medicine, University of Ulm, Albert-Einstein-Allee 23, 89081 Ulm, Germany

**Keywords:** COVID-19 pandemic, Epidemiology, Deferral, Emergency hospitalizations, Heart failure

## Abstract

**Background:**

Patients, whose non-emergency cardiac procedure was postponed during the COVID-19 pandemic, have shown signs of disease progression in the short term. Data on the long-term effects are currently lacking.

**Aim:**

To assess outcomes through 3 years following deferral.

**Methods:**

This retrospective, single-center analysis includes consecutive patients whose non-emergency cardiovascular intervention was postponed during the first COVID-19-related lockdown (March 19 to April 30, 2020). Outcomes over 36 months post-procedure were analyzed and compared to a seasonal control group undergoing non-emergency intervention in 2019 as scheduled (*n* = 214). The primary endpoint was a composite of emergency cardiovascular hospitalization and death. Additionally, NT-proBNP levels were analyzed.

**Results:**

The combined endpoint occurred in 60 of 178 patients (33.7%) whose non-emergency transcatheter heart valve intervention, rhythmological procedure, or left heart catheterization was postponed. Primary endpoint events did not occur more frequently in the study group during the 36-month follow-up (*p* = 0.402), but within the first 24 months post-procedure (HR 1.77, 95% CI 1.20–2.60, *p* = 0.003). Deferred patients affected by an event in the postprocedural 24 months had significantly higher NT-proBNP levels at the time of intervention (*p* < 0.001) (AUC 0.768, *p* = 0.003, optimum cut-off 808.5 pg/ml, sensitivity 84.2%, specificity 65.8%) and thereafter (*p* < 0.001).

**Conclusion:**

Deferral of non-emergency cardiovascular interventions is associated with poor outcomes up to 24 months post-procedure. Adverse effects affect patients who develop signs of acute heart failure, as indicated by NT-proBNP, prior to treatment. These findings could help improve resource allocation in times of limited capacity.

**Graphical Abstract:**

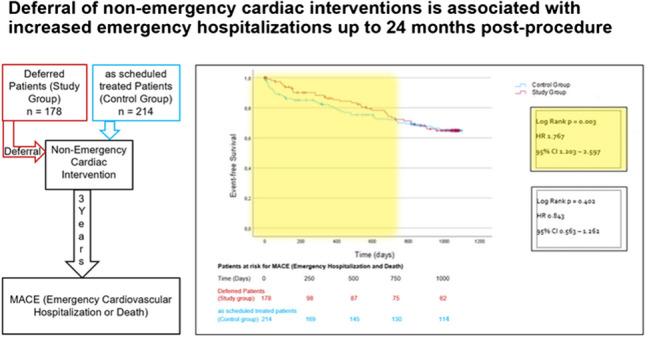

**Supplementary information:**

The online version contains supplementary material available at 10.1007/s00392-024-02380-y.

## Introduction

During the first phase of the COVID-19 crises in spring 2020, hospitals were forced to reduce their numbers of treatments to provide the capacity in anticipation of a demand surge for hospital beds from patients suffering from SARS-CoV-2 and to prevent nosocomial infections. Thus, cardiac societies have developed recommendations suggesting to cancel, postpone, or defer treatments categorized as non-emergency or elective. According to current recommendations of the European Society of Cardiology (ESC), patients were classified as deferrable in case of stable symptoms, neither recent emergency hospitalization nor critical findings [1]. Declining numbers of treatments [2, 3], especially elective appointment [4], reflected the successful implementation. However, these measures were accompanied by an increased risk for complications [4–6]. Among others, interventional cardiology was highly affected since those patients proved to be particularly vulnerable. Recent studies showed poor clinical outcomes of deferred patients in the short term up to 12 months [5, 6]. During the waiting period, affected patients developed signs of cardiac decompensation that persisted even after the procedure [5]. Patients scheduled for transcatheter aortic valve implantation showed worsening of heart failure after 31 days [6]. Outcomes were worse even 12 months after performance of treatment, as deferred patients had more often emergency hospitalizations and showed poorer cardiac status, indicated by worsened clinical symptoms and higher levels of biomarkers [5]. Data on the long-term outcomes of deferred patients are still limited. However, data on the further course of the disease could help to improve the care structures, in particular the allocation of resources, to better manage these patients. Therefore, we aimed to assess deferred patients’ outcomes through 3 years.

## Methods

### Study design

We conducted an observational case–control study including all consecutive cardiac patients whose non-emergency appointment at the Department of Medicine II at Ulm University Medical Center, Ulm, Germany, was deferred during the first COVID-19-related lockdown between March 19, 2020, and April 30, 2020 (study group). Patients scheduled for cardiac intervention due to (1) severe valve stenosis or regurgitation, (2) suspected or known significant coronary artery disease, (3) atrial or ventricular arrhythmia, or (4) implantable cardioverter defibrillator and permanent pacemaker were eligible for this study. All patients in the study group were classified as “non-emergency” or “elective” and thus considered to be deferrable according to the current recommendations of the European Society of Cardiology (ESC) [1]. Only patients who later underwent the intended intervention were included in the analyses. Non-emergency cardiac patients admitted during the corresponding period of the previous year (March 19 to April 30, 2019) served as a control group.

All patients gave informed consent to participate in the study. The study conforms to the guidelines of the Declaration of Helsinki, adheres to the STROBE statement, and was approved by the local ethics committee (ethics application 252/20).

### Data collection, follow-up, and laboratory procedures

Demographic, clinical, and laboratory data at baseline, at the time of the actual intervention performed and at follow-up, as well as outcome data, were extracted from our patient management system by two physicians and adjudicated by a third one in case of any kind of difference. Missing data were complemented by telephone interviews. Patients were scheduled for outpatient clinic visits including clinical assessment and focused cardiovascular examinations such as 12-lead ECG, transthoracic echocardiography, and laboratory blood tests at 1, 3, and 6 months after the procedure and thereafter every 6 months, as part of our clinical routine, whenever possible. Symptoms were classified according to the NYHA (New York Heart Association), EHRA (European Heart Rhythm Association), and CCS (Canadian Cardiovascular Society) scales. Left ventricular systolic function was measured either by echocardiography (EPIQ 7, Koninklijke Philips N.V., Eindhoven, Netherlands) or cardiac ventriculography during cardiac catheterization and categorized as normal, mildly impaired, moderately impaired, or severely impaired, according to guideline-specific recommendations [7]. Blood samples were drawn to measure highly sensitive cardiac troponin T (hs cTnT), NT-proBNP, and creatinine (ElectroChemiLumineszenz ImmunoAssay “ECLIA” Roche, Cobas 8000, Modul e801 and e601).

### Primary and secondary endpoints

The primary endpoint was a composite of emergency cardiovascular hospitalization or death. Additionally, the occurrence of each of these events was analyzed separately. Secondary endpoints were plasma NT-proBNP levels. The observation period was up to 36 months after the intervention.

### Statistical analysis

Continuous variables were presented as mean ± standard deviation or median together with the interquartile range (IQR) as appropriate. The Kolmogorov–Smirnov-test was used to assess normal distribution of continuous parameters. If a metric variable was not normally distributed at any date of measurement, all values were presented as median together with the interquartile range (IQR). For some variables (NYHA class, CCS class, LVEF), the mean value with standard deviation was given for reasons of clarity and comprehensibility. Categorial variables were described as number and percentage. Student’s *t*-test, Mann–Whitney *U*-test, or chi^2^ test were used to compare variables between groups where appropriate. The Kaplan–Meier estimator was used to assess the time to first event, and a comparison of groups was performed using the Cox proportional hazard model. A binary regression analysis was performed to examine the predictive value of NT-proBNP for a primary endpoint event. The incidence rates were shown as hazard ratio (HR) with 95% confidence interval (CI). Receiver operating characteristic (ROC) analysis was performed to assess the performance of the model by estimating sensitivity, specificity, and area under the curve (AUC). The Youden Index was used to calculate the optimal cut-off value for predicting the occurrence of a primary endpoint event.

Parameters with a* p* value < 0.05 were considered statistically significant. Statistical analysis was performed using SPSS Statistics 27 software (Version 2020, IBM, Armonk, NY, USA).

## Results

### Study population

Non-emergency cardiac interventions of 193 patients were deferred at our tertiary care center between March 19, 2020, and April 30, 2020 (study group). Fifteen patients were excluded because planned intervention was not performed until the end of the follow-up period. All remaining 178 patients were analyzed. Of these, 74 patients (41.6%) underwent cardiac catheterization, 49 patients (27.5%) transcatheter heart valve interventions, and in 55 patients (30.9%) a rhythmological cardiac procedure was performed, including 47 (26.4%) with electrophysical procedure and 8 (4.5%) with device implantation. In the reference period between March 19, 2019, and April 30, 2019, 216 patients (control group) were scheduled for such a cardiac intervention. Thereof, 214 patients were included as they received the planned intervention regularly. Of these, 93 patients (43.5%) underwent cardiac catheterization, 47 patients (22.0%) heart valve intervention, and 74 patients (34.6%) a rhythmological cardiac intervention, including 57 patients (26.6%) with electrophysical procedure and 17 (7.9%) with device implantation.

The baseline data of both groups are shown in Table 1. Median age did not differ between the groups (study group 72.31 ± 13.19, control group 70.29 ± 11.18 years, *p* = 0.105) and the majority of patients in both groups were male (study group 105 of 178, 59%, control group 142 of 214, 66%, *p* = 0.142). Significantly more patients in the control group had a positive family history for cardiovascular disease (study group 20 of 145, 14%, control group 57 of 214, 27%, *p* = 0.004). Furthermore, EHRA class was significantly higher in the control group (study group 1.4 ± 0.6, control group 1.4 ± 0.8, *p* = 0.006). In the study group, there were more patients with known coronary artery disease (study group 112 of 146, 77%, control group 140 of 214, 65%, *p* = 0.026). Moreover, mean heart rate was significantly higher than in the control group (study group 73 (64, 85) bpm, control group 68 (60, 80) bpm; *p* = 0.014). Additionally, those patients had significantly higher troponin T levels at baseline (study group 22.0 (10.75, 36.5), control group 15.5 (9, 28), *p* = 0.030). No further significant differences were observed in the baseline patient characteristics, especially NT-proBNP levels at the time of the originally planned intervention were similar (*p* = 0.995).

**Table 1:** Baseline characteristics

	Study group	Control group	*p* value
	*n* = 178	*n* = 214	
Age (years)	72.31 ± 11.18	70.29 ± 13.19	0.105
Male sex (%)	105 (59)	142 (66)	0.142
Height (cm)	172.50 ± 9.45	171.27 ± 9.29	0.373
Weight (kg)	86.14 ± 19.07	83.79 ± 17.88 (158)	0.385
Heart rate (bpm)	73 (64, 85)	68 (60, 80)	**0.014**
Blood pressure systolic (mmHg)	129.69 ± 19.82	129.20 ± 20.20	0.921
Blood pressure diastolic (mmHg)	75.94 ± 14.40	78.92 ± 12.74	0.390
Arterial hypertension (%)	114 (78)	171 (80)	0.693
Dyslipidemia (%)	91 (63)	154 (72)	0.083
Diabetes mellitus (%)	45 (31)	50 (23)	0.114
Family history (%)	20 (14)	57 (27)	**0.004**
Smoker (%)	52 (36)	80 (37)	0.824
Obesity (%)	32 (22)	54 (25)	0.530
History of TIA/stroke (%)	17 (12)	17 (8)	0.271
COPD (%)	13 (9)	13 (6)	0.407
OSAS (%)	10 (7)	11 (5)	0.648
CKD (%)	28 (19)	36 (17)	0.577
Known CAD (%)	112 (77)	140 (65)	**0.026**
Known cardiac arrhythmia (%)	89 (61)	121 (57)	0.402
Planned valve intervention (%)	49 (28)	47 (22)	0.238
Planned rhythmological intervention (%)	55 (31)	74 (35)	0.452
Planned left heart catheterization (%)	74 (42)	93 (43)	0.758
Percutaneous coronary intervention	71 (95.9)	87 (93.5)	0.498
NYHA class	1.9 ± 0.8	1.9 ± 0.8	0.402
EHRA class	1.4 ± 0.6	1.4 ± 0.8	**0.006**
CCS class	0.7 ± 1.1	0.8 ± 1.2	0.130
Troponin T (ng/l)	22.0 (10.75, 36.5)	15.5 (9, 28)	**0.030**
NT-proBNP (pg/ml)	866 (229, 1963)	538.5 (157.75, 1569.25)	0.995
Creatinine (µmol/l)	104.71 ± 53.36	105.80 ± 91.18	0.922
LVEF	2.3 ± 1.1	2.1 ± 1.2 (194)	0.108

*bpm*, beats per minute; *COPD*, chronic pulmonary obstructive disease; *OSAS*, obstructive sleep apnea syndrome; *CKD*, chronic kidney disease; *CAD*, coronary artery disease; *NYHA*, New York Heart Association; *CCS*, Canadian Cardiovascular Society; *LVEF*, left ventricular ejection fraction; values are shown as mean ± SD, median [IQR], or as number (%); significant differences are presented in bold

The mean time to follow-up of event-free patients was 923 days in the study group and 1095 days in the control group after the actual intervention date. The intervention of the deferred patients of the study group was postponed by a mean of 23 (19, 36.5) days. The waiting time of patients affected by MACE was 21 (16.5, 34.5) days, of event-free patients 24 (20, 45.5) days, which was not a significant difference (*p* = 0.106).

### Outcome through the subsequent 36 months: cardiovascular hospitalization and mortality

**Fig. 1:**
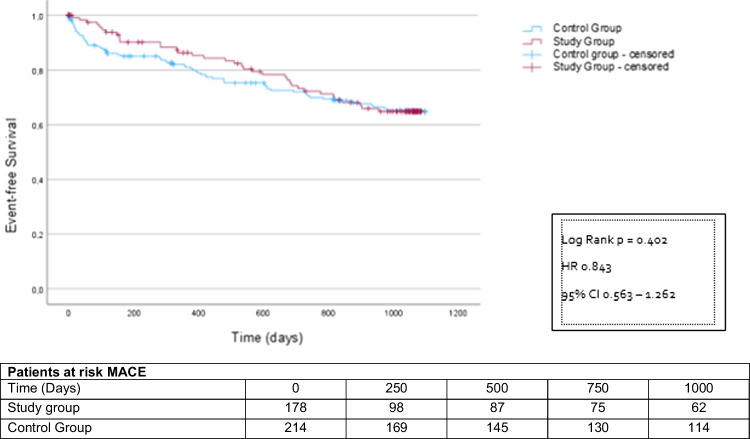
Kaplan–Meier analysis of the time to emergency hospitalization and death starting at the time of the deferred intervention, follow-up period 3 years

The Kaplan–Meier event rates at the end of the 36-month follow-up period were not significantly different between both groups. Neither the composite primary endpoint events (study group 60 patients, 33.7%, control group 69 patients, 32.2%; HR 0.84 CI 0.56–1.26; *p* = 0.402 (Fig. 1) nor the isolated event rates of emergency hospitalization (study group 52 patients (29.2%), control group 60 patients (28.0%); HR 0.77 CI 0.50–1.21; *p* = 0.26) (Supplementary Fig. 1) or death (study group 8 patients (4.5%), control group 10 patients (4.7%); HR 1.36 CI 0.54–3.44; *p* = 0.535) (Supplementary Fig. 2) differed at this time point.

**Fig. 2:**
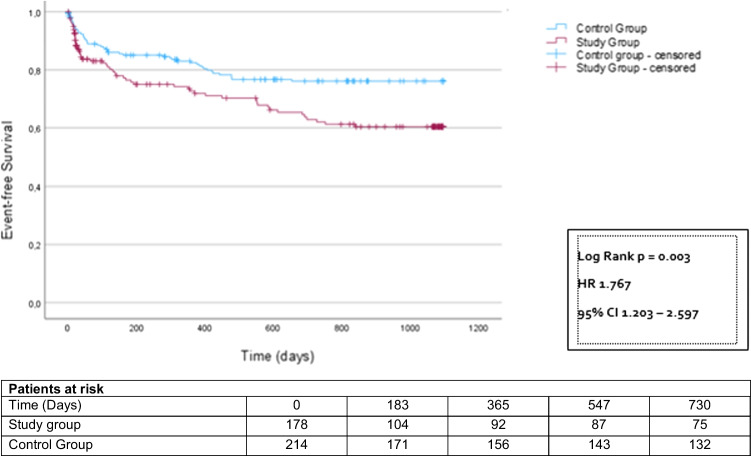
Kaplan–Meier analysis of the time to emergency hospitalization and death starting at the time of the deferred intervention, follow-up period 2 years

**Fig. 3:**
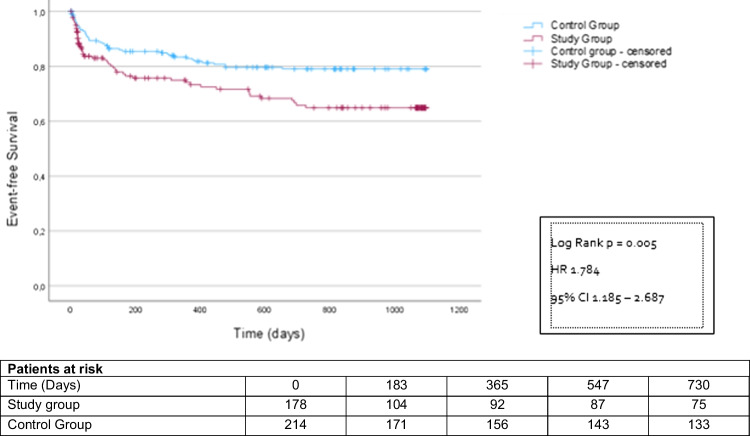
Kaplan–Meier analysis of the time to emergency hospitalization starting at the time of the deferred intervention, follow-up period 2 years

**Fig. 4:**
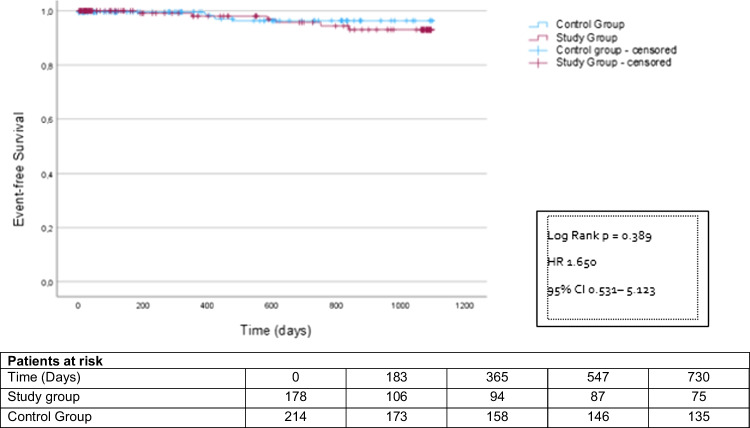
Kaplan–Meier analysis of the time to death starting at the time of the deferred intervention, follow-up period 2 years

However, after 24 months, significantly more patients in the study group had reached the combined endpoint compared to the control group (study group 57 patients (32.0%), control group 48 patients (22.4%); HR 1.77 CI 1.12–2.60; *p* = 0.003) (Fig. 2). This was driven by more emergency hospitalizations (study group 51 patients (28.7%), control group 42 patients (19.6%); HR 1.78 CI 1.19–2.69; *p* = 0.005) (Fig. 3). All-cause mortality did not differ between the groups (study group 6 patients (3.4%), control group 6 patients (2.8%); HR 1.65 CI 0.53–5.12; *p* = 0.389) (Fig. 4). MACE rates were particularly higher in postponed patients undergoing heart valve procedure compared to the corresponding regularly treated controls (44.9% vs. 23.4%; *p* = 0.027). Subgroup analyses investigating the incidences of MACE within 24 months post-procedure depending on the underlying cardiac disease are displayed in Supplementary Table 1.

### NT-proBNP levels and their correspondence with poor outcomes following deferral

#### NT-proBNP levels of event-free patients 36 months post-procedure

To further evaluate the long-term effects of deferral, outcomes from patients with no primary endpoint event were assessed in more detail. NT-proBNP plasma concentrations at the end of the 36-month follow-up period, indicating cardiac status, were compared between the groups. At that time, mean NT-proBNP levels of the deferred and regularly treated patients did not differ significantly (study group 456 (131, 1339.25) pg/ml, control group 390 (129.25, 1378.25) pg/ml, *p* = 0.885) (Table 2).

**Table 2:** NT-proBNP levels (pg/ml) of patients without MACE in the study group and control group

	Study group	Control group	*p* value
	*n* = 121	*n* = 166	
Baseline	710 (166.5, 1642)	454.5 (143, 1547)	0.960
Admission	496 (164.25, 1675)	454.5 (143, 1547)	0.952
12 months follow-up	440 (149.5, 1070)	380 (86.5, 786)	0.803
36 months follow-up	456 (131, 1339.25)	390 (129.25, 1378.25)	0.885

Values are shown as median [IQR]

#### Predictive value of NT-proBNP levels for poor outcomes following deferral

**Fig. 5:**
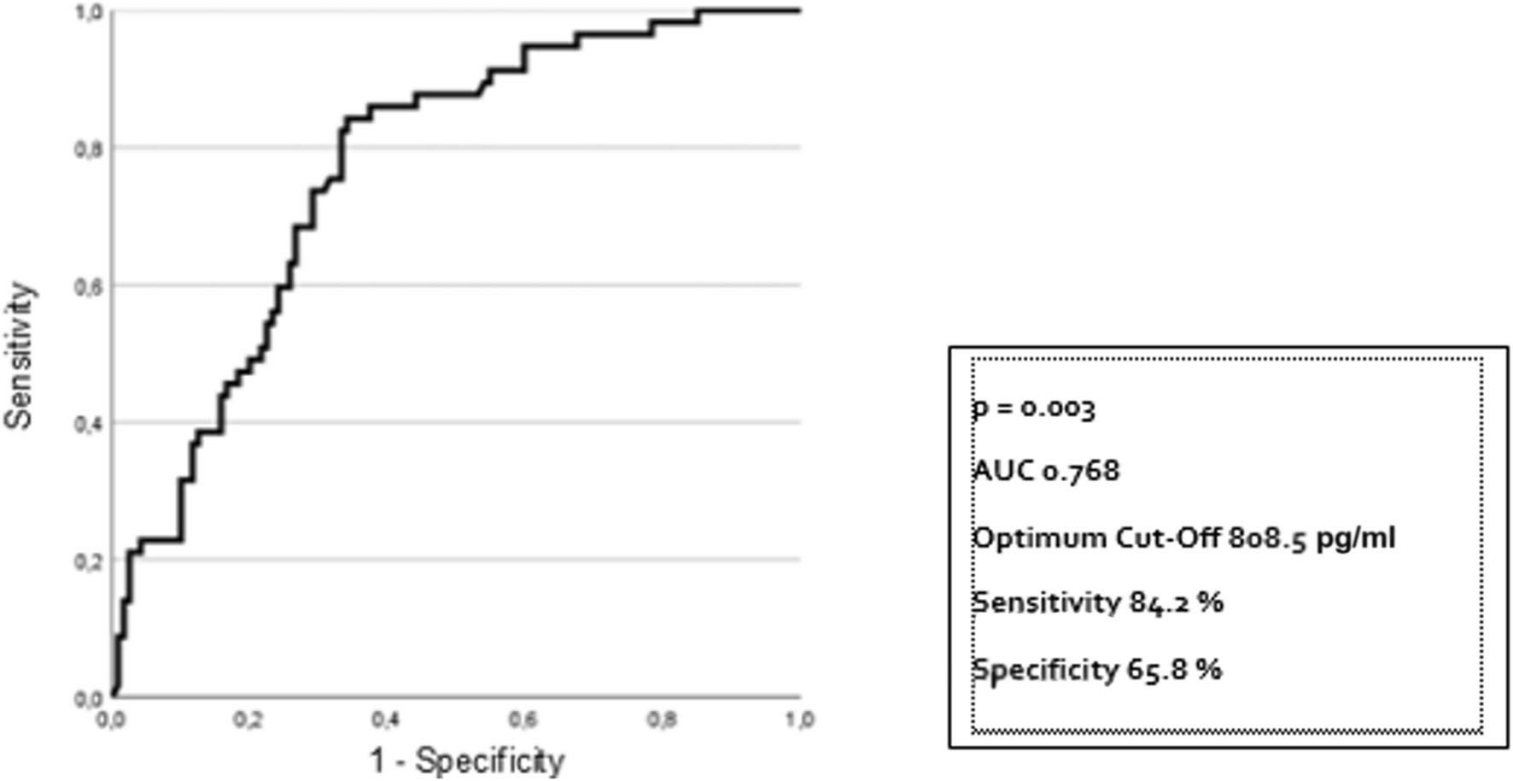
Predictive value of NT-proBNP at admission for emergency hospitalization and death within 24 months following the intervention by receiver operating characteristic (ROC) curve

NT-proBNP has already been proven to be a suitable risk indicator for the current cohort, predicting poor outcomes, including primary endpoint event rates, at 12 months [8]. Since the period of increased risk due to deferral has been identified, we aimed to assess the predictive value of NT-proBNP levels for primary endpoint events during this period. For this purpose, the endpoint was defined as an event of emergency cardiovascular hospitalization or death within this high-risk period of 24 months post-procedure. Binary logistic regression analysis revealed that high levels of NT-proBNP are associated with an increased risk (HR 1.77, 95% CI 1.20–2.60; *p* = 0.003). The ROC analysis yielded an area under the curve of 0.768. The optimal cut-off was 808.5 pg/ml, which had a sensitivity of 84.2% and a specificity of 65.8% (Fig. 5). Among patients with NT-proBNP levels equal or greater than this cut-point, the Kaplan–Meier event rate for the primary endpoint was 53.93% (48 of 89 patients), in patients with lower levels 10.11% (9 of 89 patients). In contrast, in the control group, NT-proBNP levels at admission were not predictive of the occurrence of a primary endpoint event within the following 24 months (*p* = 0.208).

#### Comparison of NT-proBNP levels of patients with and without primary endpoint events over time

Since NT-proBNP was shown to reflect the risk of adverse outcomes, the levels of this biomarker were analyzed at different time points to further investigate the impact of delayed treatment on patients’ disease progression. For this purpose, patients were divided into subgroups based on the occurrence of a primary endpoint event within 24 months after the intervention. Deferred patients affected by such an event within this period had significantly higher mean NT-proBNP levels than event-free patients at the time of admission to the intervention (event group 1933 (1038, 4203) pg/ml, event-free group 496 (164.25, 1675) pg/ml, *p* < 0.001) and also at the 12 months follow-up (event group 2779 (1094.50, 5877) pg/ml event-free group 440 (149.5, 1070) pg/ml, *p* < 0.001). However, no such difference was observed before waiting time (event group 1544 (813, 3726) pg/ml, event-free group 710 (166.5, 1642) pg/ml, *p* = 0.224) (Table 3). Additionally, NT-proBNP levels at admission in patients affected by a primary endpoint event within 24 months following the procedure were significantly higher in the study group compared to the control group (study group 1933 (1038, 4203) pg/ml, control group 846 (243.5, 1756.75), *p* < 0.001) (Table 4). In contrast, NT-proBNP levels at admission of event-free patients were similar in the control and the study group (study group 496 (164.25, 1675) pg/ml, control group 454.5 (142.25, 1534.75) pg/ml; *p* = 0.952) (Table 2). Moreover, in the control group, NT-proBNP levels at baseline and admission were similar in patients with and without a primary endpoint event in the following 24 months (*p* = 0.183) (Table 5).

**Table 3:** NT-proBNP levels (pg/ml) of deferred patients (study group) with and without MACE

	Patients with MACE	Patient without MACE	*p* value
	*n* = 57	*n* = 121	
Baseline	1544 (813, 3726)	710 (166.5, 1642)	0.224
Admission	1933 (1038, 4203)	496 (164.25, 1675)	** < 0.001**
12 months follow-up	2779 (1094.5, 5877)	440 (149.5, 1070)	** < 0.001**

Values are shown as median [IQR], significant differences are presented in bold

**Table 4:** NT-proBNP levels (pg/ml) of patients with MACE in the study group and control group

	Study group	Control group	*p* value
	*n* = 57	*n* = 48	
Baseline	1544 (813, 3726)	846 (243.5, 1756.75)	0.990
Admission	1933 (1038, 4203)	846 (243.5, 1756.75)	** < 0.001**
12 months follow-up	2779 (1094.5, 5877)	705.5 (301.25, 2278.25)	**0.006**

Values are shown as median [IQR], significant differences are presented in bold

**Table 5:** NT-proBNP levels (pg/ml) of regularly treated patients (control group) with and without MACE

	Patients with MACE	Patients without MACE	*p* value
	*n* = 48	*n* = 166	
Baseline	846 (243.5, 1756.75)	454.5 (143, 1547)	0.183
Admission	846 (243.5, 1756.75)	454.5 (143, 1547)	0.183
12 months follow-up	705.5 (301.25, 2278.25)	380 (86.5, 786)	**0.043**

Values are shown as median [IQR], significant differences are presented in bold

## Discussion

In this study, we analyzed the outcomes of patients whose non-emergency cardiac interventions were postponed in course of the first COVID-19-related lockdown. In these patients, we observed an increased risk of emergency hospitalization up to 24 months after the scheduled intervention was performed. Affected patients developed high NT-proBNP levels prior to treatment indicating cardiac impairment. Remarkably, these high values persisted post-procedure suggesting ongoing cardiac damage. In contrast, we did not observe negative effects of deferral in patients with stable concentrations of NT-proBNP.

During the first wave of the COVID-19 pandemic in spring 2020, a reduction of hospital admissions was sought in order to provide the capacity for the expected increased demand surge by SARS-CoV-2 infections. Therefore, cardiac societies developed strategies to identify patients in whom non-emergency procedures can safely be deferred [1, 9, 10]. Despite classified as deferrable, poor short-term outcomes have been observed in affected patients [5, 6]. The AS-DEFER study reported worsening of heart failure in deferred patients scheduled for aortic valve replacement due to severe symptomatic aortic valve stenosis 31 days after treatment strategy assignment. Deferred patients more often experienced disease-related hospitalizations [6]. A recent study including a large variety of cardiac patients observed poorer outcomes in deferred patients even after 12 months. Non-emergency cardiac patients scheduled for transcatheter valve intervention, left heart catheterization, or rhythmological procedures including ablation of arrhythmia and device implantation showed an increased rate of emergency hospitalizations substantiated by more pronounced symptoms and higher levels of biomarkers, whereas regularly treated patients showed an improvement of symptoms [5]. However, at present, intermediate and long-term outcomes remain unclear due to lack of experience.

### Outcomes: deferred patients show higher rates of emergency hospitalization up to 24 months after treatment

Major adverse cardiac events (MACE) are a well-established endpoint for assessing outcomes of cardiac patients and have been widely used to assess the impact of the COVID-19 pandemic on this collective [11], even for the cohort of patients with deferred non-emergency cardiac procedures [5].

In the current study, we could not detect any differences in the combined endpoint of emergency hospitalizations and death between deferred and regularly treated patients after 36 months. Because outcomes after 12 months have been reported to be poor [5, 6], we analyzed the 24-month outcomes to enclose the period of increased risk. In our study, 24 months after the performance of the intervention, deferred patients still showed poorer outcomes as they had significantly more endpoint events, which was mainly driven by emergency hospitalizations. This suggests that the period of increased risk of poor outcomes after deferral lasts up to 24 months post-procedure.

### Outcomes: NT-proBNP and risk of adverse outcomes

The getABI study found a significant association of NT-proBNP with cardiovascular and all-cause mortality, suggesting an independent risk prediction [12]. These data were confirmed by a further analysis, which found a predictive value of NT-proBNP according to mortality and multiple adverse cardiovascular events [13]. Moreover, BNP/NT-proBNP was found to be the best independent predictor of cardiovascular mortality, as it identifies any form of asymptomatic target organ damage (TOD), even in an early, silent stage. The strong evidence that it may be able to identify impending TOD in a few years [14] demonstrates its predictive potential. Consistent with this, BNP and NT-proBNP are most commonly used in diagnosis and follow-up of patients with acute heart failure [15]. Moreover, NT-proBNP has been approved to be suitable for assessing risk of MACE in deferred patients [6], even in the present cohort [5, 8]. In the present study, patients not experiencing emergency hospitalization or death within 36 months of follow-up showed stable concentrations of NT-proBNP levels indicating an overall stable condition.

### Outcomes: timeline of adverse outcomes

We found an increased risk of adverse outcomes up to 24 months after the deferred procedure. An investigation of the course of cardiac rehabilitation over time showed that 85% of the predicted recovery was achieved in the first 10 to 21 weeks in patients undergoing their intended interventions regularly [16]. Another study on rehabilitation time after myocardial infarction and coronary artery bypass graft suggests even faster recovery, as the results of a 4-week and 10-week course did not differ [17]. This is reflected in the recommendations for cardiac monitoring suggesting reassessment of cardiac state after acute myocardial injury after 6 to 12 weeks. For example, reassessment of left ventricular ejection fraction recovery after acute myocardial infarction, which is required for risk stratification in the decision on an implantable defibrillator (ICD), should be done 6 to 12 weeks after revascularization. In the absence of intervention, annual or biannual reassessment to monitor cardiac disease progression is recommended [11, 18, 19]. In the present study, in contrast to the expected cardiac recovery, negative effects due to deferral persisted even for a long time after the performance of the intended cardiac intervention.

### Patients at risk: increased NT-proBNP levels after the waiting time are associated with poor outcomes of deferred patients

In a study of patients with ST-elevation myocardial infarction (STEMI) undergoing percutaneous coronary intervention (PCI), a positive correlation between NT-proBNP and MACE within 1 month was observed [20]. Previous analysis of the present collective yielded comparable results, with MACE being predicted by elevated NT-proBNP levels after the waiting time [5, 8]. This raises the question of whether the performance of a procedure even after deferral may reverse myocardial damage.

In our study, NT-proBNP levels at the time of admission to the deferred intervention predict the occurrence of emergency hospitalization during the time at risk indicating additional myocardial damage during the waiting period. This suggests that outcomes are determined prior to treatment and are not completely reversable. We calculated a threshold of 808.5 pg/ml corresponding to previously described cut-points of > 900 pg/ml for accurately identifying acute heart failure in the age group of 70 to 75 years [21], similar to the mean age of the patients in our study. Moreover, it is comparable to the cut-point calculated in the 12 months outcomes analysis of this cohort [8]. This strengthens the hypothesis that poor outcomes following deferral of cardiac interventions are mainly due to pre-procedure acute heart failure.

### Consequence: rapid progression of cardiac diseases requires timely treatment

In summary, the present findings suggest that postponing non-emergency cardiac interventions in patients at risk may induce permanent myocardial damage and incidental acute heart failure during the waiting period. This emphasizes the need for timely treatment of patients with rapid progression of cardiac disease. NT-proBNP might be very useful to identify patients at risk for acute heart failure during the waiting period. Increasing concentrations of NT-proBNP might help to determine the suitable time of treatment.

## Limitations

The present analysis is a monocentric, retrospective observational study carrying all the inherent limitations ascribed to such type of design. However, all consecutive patients in the defined time period were included without patient exclusion or preselection aiming to reduce selection bias as much as possible. Due to the exploratory character of the study, the results have to be interpreted as generating hypothesis. The definition of the inclusion criterion “non-emergency” or “elective” might also be a subject for debate, since the recommendations vary depending on the publishing society. However, deferral was indicated based on the current recommendations of the local ethics committee and the European Society of Cardiology (ESC) [1]. Next, the heterogenous collective of cardiac patients included may impair comparability; to address this, differences in the distribution of the type of cardiac disease between the investigated groups were excluded. Moreover, the broad collective of different cardiac patients in the cohort of deferred patients may lead to the results being driven by individual subgroups. Nevertheless, we included all consecutive cardiac patients classified as non-emergency in this study to obtain an unbiased view of this population. Lastly, results of subgroup analyses investigating MACE rates depending on the underlying cardiac disease must be viewed with caution given the overall rather small patient cohort. For this reason, these analyses have been included only to the supplementary material.

## Conclusion

Deferral of non-emergency cardiac interventions is associated with an increased risk of emergency cardiovascular hospitalization within 24 months after the procedure. Affected patients showed elevated NT-proBNP levels prior to treatment. Therefore, the timely performance of non-emergency cardiac interventions seems to be necessary to avoid severe cardiac damage.

### Supplementary information

Below is the link to the electronic supplementary material.

Supplementary file1 (DOCX 58.0 KB)

## Data Availability

The data underlying this article will be shared on reasonable request to the corresponding author.
