# Supplementary material for: Deferral of non-emergency cardiac interventions is associated with increased emergency hospitalizations up to 24 months post-procedure
Source: Clin Res Cardiol. 2024 Mar 6;113(7):1041–50. doi: 10.1007/s00392-024-02380-y (PMC11219456; doi:10.1007/s00392-024-02380-y)
Supplement: Supplementary file 1 — Supplementary file1 (DOCX 58.0 KB) [file 392_2024_2380_MOESM1_ESM.docx]

Supplementary Fig. 1: Kaplan-Meier analysis of the time to emergency hospitalization starting at the time of the deferred intervention, follow-up period 3 years


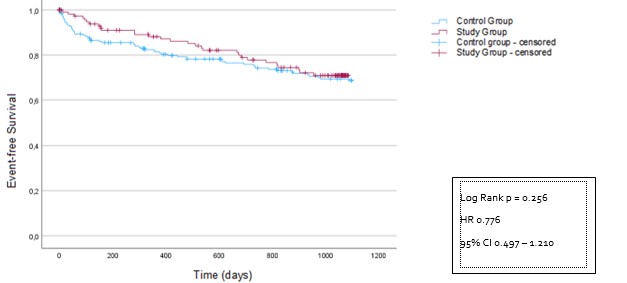


| Patients at risk |  |  |  |  |  |
| --- | --- | --- | --- | --- | --- |
| Time (Days) | 0 | 250 | 500 | 750 | 1000 |
| Study group | 178 | 98 | 87 | 75 | 62 |
| Control Group | 214 | 169 | 145 | 130 | 114 |

Supplementary Fig. 2: Kaplan-Meier analysis of the time to death starting at the time of the deferred intervention, follow-up period 3 years


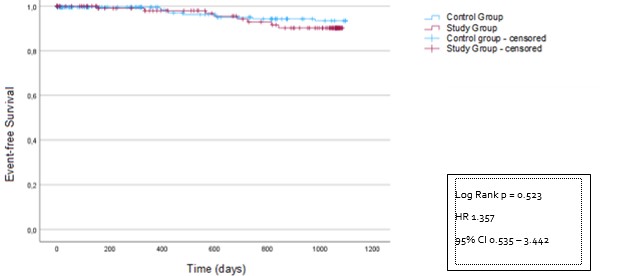


| Patients at risk |  |  |  |  |  |
| --- | --- | --- | --- | --- | --- |
| Time (days) | 0 | 250 | 500 | 750 | 1000 |
| Study group | 178 | 100 | 87 | 75 | 62 |
| Control Group | 214 | 171 | 147 | 133 | 116 |

Supplementary table 1: MACE depending on the index disease starting at the time of the deferred intervention, follow-up period 2 years

|  | | Study Group | | Control Group | | p-value | | | |
| --- | --- | --- | --- | --- | --- | --- | --- | --- | --- |
| Cardiac catheterization | | 23 (31.1) | | 22 (23.7) | | 0.290 | | | |
| Rhytmological procedure | | 12 (21.8) | | 15 (20.3) | | 0.833 | | | |
| Heart valve intervention | | 22 (44.9) | | 11 (23.4) | | 0.027 | | | |
| Aortic valve replacement | | 12 (42.9) | | 8 (24.2) | | 0.132 | |  |  |
| Values are shown as number (%), significant p-values are presented in bold | | | | | | | |  |  |
